# Mitochondria in colorectal cancer stem cells - a target in drug resistance

**DOI:** 10.20517/cdr.2022.116

**Published:** 2023-05-06

**Authors:** Mateus de Almeida Rainho, Priscyanne Barreto Siqueira, Ísis Salviano Soares de Amorim, Andre Luiz Mencalha, Alessandra Alves Thole

**Affiliations:** ^1^Laboratory of Stem Cell Research, Histology and Embryology Department, Roberto Alcantara Gomes Biology Institute, State University of Rio de Janeiro, Rio de Janeiro 20550-170, Brazil.; ^2^Laboratory of Cancer Biology, Biometry and Biophysics Department, Roberto Alcantara Gomes Biology Institute, State University of Rio de Janeiro, Rio de Janeiro 20550-170, Brazil.

**Keywords:** Cancer stem cells, mitochondria, colorectal cancer, drug resistance, mitophagy

## Abstract

Colorectal cancer (CRC) is the third most diagnosed cancer and the second most deadly type of cancer worldwide. In late diagnosis, CRC can resist therapy regimens in which cancer stem cells (CSCs) are intimately related. CSCs are a subpopulation of tumor cells responsible for tumor initiation and maintenance, metastasis, and resistance to conventional treatments. In this scenario, colorectal cancer stem cells (CCSCs) are considered an important key for therapeutic failure and resistance. In its turn, mitochondria is an organelle involved in many mechanisms in cancer, including chemoresistance of cytotoxic drugs due to alterations in mitochondrial metabolism, apoptosis, dynamics, and mitophagy. Therefore, it is crucial to understand the mitochondrial role in CCSCs regarding CRC drug resistance. It has been shown that enhanced anti-apoptotic protein expression, mitophagy rate, and addiction to oxidative phosphorylation are the major strategies developed by CCSCs to avoid drug insults. Thus, new mitochondria-targeted drug approaches must be explored to mitigate CRC chemoresistance via the ablation of CCSCs.

## INTRODUCTION

Colorectal cancer (CRC) is the third most diagnosed cancer worldwide, the second leading cause of cancer death in women, and the third in men^[1]^. Currently, the chemotherapy regimens for CRC consist of 5-fluorouracil (5-FU) plus leucovorin for early stages (stages 0, I, and II), and 5-FU plus oxaliplatin (FOLFOX) or 5-FU plus irinotecan (FOLFIRI), which may be accompanied by targeted therapies (e.g., bevacizumab or cetuximab), as a first-line treatment for more advanced stages (stages III and IV)^[2]^. FOLFOX and FOLFIRI chemotherapy regimens achieve objective response rates of approximately 50% and the overall CRC patients present 5-year survival rates of 60%. Although there are tools for genetic screening of CRC for defective DNA mismatch repair (MMR) using immunohistochemistry and/or microsatellite instability test^[3]^, diagnosis of disease at a late stage (stage IV) is frequent in CRC, which reduces the overall 5-year survival rate to approximately 18%^[1]^. In addition, nowadays, it is known that miRNAs play an important role in resistance to the chemotherapy regimens mentioned above, which makes them a relevant target for major studies^[4]^.

Regarding therapy resistance, researchers have sought new strategies, such as using CRC patient-derived organoids (PDOs), to understand this challenging problem. Recent studies suggest that PDOs can prevent patients from undergoing ineffective chemotherapy and can help to develop effective and personalized therapies against CRC^[5,6]^. However, drug resistance is still a problem to be fought, with cancer stem cells (CSCs) emerging as an important factor in the process^[7]^. CSCs are a subset of tumor cells defined by their ability to self-renewal and differentiate into distinct progenies, being also responsible for invasion and migration, leading to metastasis, and a slow-cycling cellular turnover, making them resistant to therapies^[8,9]^. CSCs have been described in many types of cancer, and colorectal cancer stem cells (CCSCs) are considered the major cause of therapeutic failure and resistance to CRC treatments, leading to tumor progression, recurrence, and, eventually, patient death^[10]^.

The mechanisms underlying CCSCs chemoresistant phenotype include reversible quiescent state, high expression of drug efflux pumps, activation of protumoral signaling pathways, such as Hedgehog, Notch, Wnt/β-catenin, Hippo, PI3K/Akt, and TGF-β, avoidance of chemotherapeutic-induced DNA damage, dysregulation of microRNAs, hypoxia microenvironment, metabolic switch for oxidative phosphorylation (OXPHOS), among others^[2]^.

Since conventional anticancer therapies act on highly proliferating cells, it does not affect quiescent cancer stem cells, as they are restrained in the G0 phase from the cell cycle, a non-proliferative state. Additionally, these cells can re-enter the cell cycle after undergoing chemotherapy, leading to cell proliferation and tumor regeneration^[11]^. Francescangeli *et al.*^[12]^ showed a high proportion of quiescent and chemoresistant CCSCs in response to treatment with oxaliplatin and 5-fluorouracil in a xenograft model.

Several studies have shown that mitochondria are involved in the mechanisms of chemoresistance of cytotoxic drugs or radiotherapy due to the many metabolic pathways conferring tumor cells’ resistant phenotype and, specifically, CSCs^[13-15]^. Therefore, it is important to understand the mitochondrial role in CRC drug resistance mediated by cancer stem cells.

## MITOCHONDRIAL ROLE IN DRUG RESISTANCE: AN OVERVIEW

Beyond the powerhouse of the cell, mitochondria have been shown to act in many different frontlines, such as cell survival, proliferation, autophagy, and calcium homeostasis^[16]^. In cancer, these double-membrane organelles play a pivotal role in establishing and progressing the disease, ranging from mitochondrial dynamics, biogenesis metabolism, and cell death regulation. Reactive oxygen species (ROS) balance mitochondria DNA (mtDNA) mutations^[17]^, drug resistance, and mitochondria work like stress sensors, mediating adaptations in the face of an adverse environment caused by chemo or radiotherapies^[18,19]^.

Some major mechanisms for drug resistance include alterations in drug transport and metabolism, enhanced DNA damage repair machinery, and inhibition of apoptosis^[20]^. Most chemoresistant tumors present a high expression of drug efflux membrane transporters, wherein the ATP-binding cassette (ABC) transporter family is the most important, highlighting ABCB1 and ABCG2, which are key players in cancer chemoresistance as they are capable of transporting the majority of conventional chemotherapeutic agents^[21]^ Since ABC transporters depend on ATP hydrolysis to pump chemotherapeutics, mitochondria have their importance heightened as they are major producers of ATP via OXPHOS. A recent study using chemoresistant ovarian cancer cells showed that electron transport chain inhibitors increased drug retention and reverted the chemoresistant phenotype in vitro and *in vivo*^[22]^.

Mitochondria morphology is also an important feature of drug resistance. It is a dynamic organelle and can vary in size and copies inside cells due to fusion and fission, which are regulated by a GTPase family of proteins. Mitochondrial fusion, which usually occurs in nutrient deprivation and increased OXPHOS cases, implicates long interconnected tubules, forming networks. Oppositely, mitochondrial fission appears as small and fragmented mitochondria, commonly associated with cellular and mitochondrial dysfunction, severe stress, and increased proliferation^[23]^. A study using cisplatin-resistant cervical and ovarian cancer cell lines exhibited a prevalence of elongated mitochondria, similar to tubular shapes, suggesting that mitochondria fusion is required to resist the drug treatment^[24]^. Nevertheless, studies in breast cancer and acute lymphoblastic leukemia cellular lineages have demonstrated that mitochondria fission is required for metabolic adaptation favorable for protecting cells from chemotherapy agents^[25,26]^. In addition, mitochondrial fission factor (MFF) is upregulated in prostate cancer stem cells compared to the primary tumor and normal prostate cells^[27]^. A novel study with metastatic breast cancer cells shows that changes in mitochondrial dynamics, tending to a fission increase, lead to increased production of mitochondrial ROS, which retrograde response endows cells with resistance to oxidative stress and reactive oxygen species-dependent chemotherapy drugs^[28]^. Therefore, the role of mitochondrial dynamics on drug resistance is an important area for further study.

Considering that mitochondria act as a metabolic hub, these organelles are expected to have great importance in modulating therapy sensitivity, especially by controlling redox metabolism^[29]^. Resistant cancer cells usually exhibit great mitochondrial functionality, with high rates of OXPHOS. For instance, many studies have shown that OXPHOS inhibitors suppress resistance to anticancer drugs in lung adenocarcinoma, prostate cancer, melanoma, pancreatic cancer, and colon cancer^[30]^. A study showed that treating ovarian cancer cells with cisplatin might induce oxidative phosphorylation and stem cell enrichment, which can be overcome using OXPHOS inhibitors^[31]^. Fatty acids oxidation (FAO), which depends on mitochondrial enzymes, is also considered an accomplice of therapeutic resistance^[32]^, as a study demonstrated that FAO inhibition promotes chemosensitivity in breast CSCs^[33]^. Lee *et al.*^[34]^ showed that liver cancer stems cell chemoresistance depends on glutamine metabolism by mitochondria since mitochondrial ATP used in drug efflux is provided by glutamine, and its inhibition reduces efflux, decreasing chemoresistance. Increased expression of MTHFD2, a mitochondrial enzyme involved in the one-carbon metabolic pathway, was observed in gefitinib-resistant lung cancer cells with stem cell features. MTHFD2 knockdown decreased stem cell phenotype and promoted gefitinib sensitization, highlighting the MTHFD2 importance and potential as a target for anticancer therapy^[35]^. Another important pathway in which mitochondria participate in is folate metabolism, also known as 1C metabolism^[36]^. This metabolic process involves the activation and transference of one carbon-molecules to support nitrogenous bases biosynthesis, antioxidant agents’ regeneration, and amino acid balance. In this scenario, mitochondria can be responsible for chemoresistance fueling 1C metabolism, then enhancing OXPHOS. Lucas *et al.*^[37]^ observed that the activity of SHMT2, a mitochondrial enzyme involved in serine and tetrahydrofolate metabolism, ensures complex I assembly and function, thus increasing the electron transport chain activity and developing resistance.

Resistance to apoptosis is described as one hallmark of cancer, and its contribution to resistant cancer cells is remarkably relevant^[38]^. Indeed, the correct functioning of mitochondria is fundamental for cellular health. It also has an essential role in programmed cell death, and its morphology is critically important for apoptosis commitment^[39]^. Changes in the permeability of the inner mitochondrial membrane lead to the loss of the mitochondrial membrane potential allowing the release of pro-apoptotic proteins, such as B-cell lymphoma 2 (Bcl-2) family proteins (Bcl-2, Bcl-xl, Bcl-W, MCL-1, A1, and Bcl-B)^[40,41]^. Xu *et al.*^[42]^ showed that overexpression of Bcl-2 blocked apoptosis in human ovarian cancer cells. In addition, other pro-apoptotic proteins, such as apoptosis-inducing factor (AIF) and endonuclease G (Endo G), are released from a late event in apoptosis, which occurs once the cells are committed to dying^[40]^. AIF and Endo G act in a caspase-independent manner to execute cell death^[43]^. Alvero *et al.*^[44]^ were shown that Endo G mediates caspase-independent cell death in response to chemotherapeutic agents in an ovarian cancer model. One of the aims of chemotherapy is to induce apoptosis by targeting these pro-apoptotic proteins, mainly the Bcl-2 family, creating mitochondrial outer membrane permeabilization, which results in the irreversible release of cytochrome c, caspase activation and then apoptosis^[45]^. For instance, docetaxel, a common chemotherapeutic agent whose primary mechanism of action is inhibition of microtubule disassembly, can also bind to Bcl-2, inducing apoptosis^[46]^. Furthermore, a cutting-edge contribution of mitochondria in cancer drug resistance is the exchange of this organelle mediated by tunneling nanotubes, a transient cytoplasmic connection between non-adjacent cells^[47]^. Several studies have described mitochondrial transfer from important stromal cells of the tumor microenvironment, such as endothelial cells and mesenchymal stem cells, to cancer cells in many types of cancer, such as breast cancer^[48]^, acute myeloid leukemia^[49]^ and glioblastoma^[50]^. This mechanism promotes tumor survival and chemoresistance by improving OXPHOS or increasing anti-apoptotic proteins.

### Mitochondria-targeted drugs for cancer - current status

Since mitochondria are a central organelle for cell survival, including cancer cells, several researchers have dedicated time and effort in recent years to develop new anticancer drugs and strategies that could target mitochondria. This group of compounds, proposed to impair mitochondria, are designated as mitochondria-targeted drugs (MTDs). Their mechanism of action can range from targeting tricarboxylic (TCA) cycle enzymes to electron transport chain (ETC) complexes and Bcl-2 anti-apoptotic family proteins^[51]^. Some MTDs studied comprise Mito-carboxy proxyl (Mito-CP), Mito-metformin, and Mito-methyl coumarin.

Mito-CP, a lipophilic cationic nitroxide conjugated to an alkyl triphenylphosphonium cation (TPP), showed to accumulate in mitochondria, combined with 2-deoxyglucose (2-DG), a glycolysis inhibitor, in hepatocellular carcinoma and breast cancer cells, inducing apoptosis via caspase 3/7 activation and a significant decrease in intracellular ATP. Notwithstanding, Mito-CP did not affect primary hepatocytes cells or non-tumoral fibrocystic breast cell line (MCF10A), highlighting a possible selectivity of this compound^[52,53]^. Concerning colorectal cancer, Boyle *et al.*^[54]^ demonstrated for the first time that MTDs induce mitophagy in cancer cells. In the study, Mito-CP and Mito-Metformin, a TPP-conjugated derivative of a common type 2 diabetes drug metformin which acts inhibiting complex I of mitochondrial respiratory chain, released Unc-51-like autophagy-activating kinase 1 (ULK1) from mTOR-mediated inhibition, affecting mitochondrial morphology, and decreasing mitochondrial membrane potential, which are indicators of mitophagy. Another MTDs is Mito-methyl coumarin, a compound synthesized using the coumarin backbone, which exhibited anticancer proprieties in Hela cells by increasing ROS generation, reducing mitochondrial mass and membrane potential, and inducing programmed cell death^[55]^.

Regarding cancer chemotherapy resistance, Chan *et al.*^[56]^ showed that drug molecules carried by multifunctional nanodiamonds could be effectively delivered to mitochondria and induce remarkable cytotoxicity and cell death in doxorubicin-resistant MCF-7 breast cancer cells. In addition, Li *et al.*^[57]^ synthesized mitochondria-targeted polydopamine nanoparticles loaded with doxorubicin and demonstrated that this system could potentially overcome drug resistance with a short-term treatment plan toward breast cancer cell lines (MDA-MB-231).

Focusing on cancer stem cells (CSCs), MTDs are considerably more effective in triggering apoptosis of CSCs, when compared to other agents, via mitochondrial dysfunction by regulating Bcl-2 family proteins, and ROS production^[58]^. Thus, specific mitochondrial-targeted compounds can induce cell death in chemoresistant CSCs. For example, Hirsch *et al.*^[59]^ showed that metformin, an inhibitor of the complex I mitochondrial respiratory chain, selectively kills CSCs in breast cancer cell lines. Alvero *et al.*^[60]^ demonstrated that the novel isoflavone derivative NV-128 significantly decreased mitochondrial function and induced cell death in ovarian CSCs.

Although only one MTD has been approved for cancer treatment (BH3 mimetic Venetoclax) so far, other drugs have shown beneficial and promising effects in phase I/Ib and phase I clinical trials, such as MitoTam (EudraCT 2017-004441-25)^[61]^ and IACS-01075917 (NCT03291938)^[62]^, respectively. Given the importance of mitochondria for cancer cells, especially cancer stem cells, further research on agents that target mitochondria and combining these drugs with other chemotherapeutics are encouraged to increase the efficiency of anticancer therapies.

## CANCER STEM CELLS AND MITOCHONDRIA: A STRONG PAIR IN DRUG RESISTANCE IN CRC

As already described, mitochondria are involved in many metabolic pathways, and alterations in this metabolism have been found in CSCs^[24]^. Huang *et al.*^[63]^ discovered that suppressing mitochondrial ROS production drives glioma stem-like cell progression and facilitates radiotherapeutic resistance. In addition, Ren *et al.*^[64]^. observed alterations in mitochondrial function, such as the low amount of mtDNA and oxygen/glucose consumption and low intracellular ROS and ATP concentrations in thyroid cancer stem cells, which contribute to radioresistance. Kuntz *et al.*^[65]^ indicate that alterations in essential mitochondrial functions, such as oxidative phosphorylation, contribute to therapy-resistant chronic myeloid leukemia stem cells.

The damage of mtDNA, or even its loss, causes a reduction in the proliferation rate of CSCs^[27]^. Huang *et al.*^[66]^ show that mtDNA deficiency may induce ovarian cancer stem cell-like properties and reveal the downregulation of mitochondria-related genes and upregulation of genes related to cell proliferation, anti-apoptosis, and drug resistance. Although these data indicate mitochondrial dysfunction and OXPHOS reduction, it has been reported the coexistence of a CSCs subpopulation that produces ATP via an anaerobic glycolytic pathway and feeds the OXPHOS of the OXPHOS-addicted subpopulation. This metabolic shift, known as the Warburg effect, usually occurs in regions of tumor hypoxia^[67]^. Genetic and epigenetic alterations were found in the mtDNA of cisplatin-resistant oral squamous cancer cells. Further study of these changes may help to elucidate their role in chemoresistance^[68]^.

Concerning apoptosis, CSCs survival depends on the dysregulation of apoptosis pathways and the overexpression of anti-apoptotic proteins^[69]^. The anti-apoptotic proteins include the cellular FLICE-inhibitory protein (c-FLIP), the Bcl-2 family of proteins, and the inhibitor of apoptosis proteins (IAPs). Piggott *et al.*^[70]^ showed that the overexpression of c-FLIP plays a crucial role in resistance to TNF-related apoptosis-inducing ligand (TRAIL)-induced apoptosis, an anticancer agent, in breast cancer stem cells. Guo *et al.*^[71]^ indicated that survivin/BRIC5, a member of the IAPs family, is promising to be an excellent candidate for recombinant anti-cancer protein by promoting apoptosis of cancer cells and their stem cells through sensitizing cells to chemotherapeutic drugs.

Furthermore, CSCs and mitochondrial dynamics are valuable partners for overcoming drug resistance^[72]^. Brain CSCs derived from tumor xenografts and primary tumor samples showed higher fragmented mitochondria and activation phosphorylation of dynamin-related protein 1 (DRP1) compared to non-CSCs^[73]^. Notwithstanding, in a breast cancer model, mitochondrial fusion promoted by upregulation of MCL-1 showed to maintain CSCs properties in cooperation with MYC, facilitating mitochondrial respiration, thus inducing chemotherapy resistance^[74]^.

### Apoptotic proteins expression

**Figure 1:**
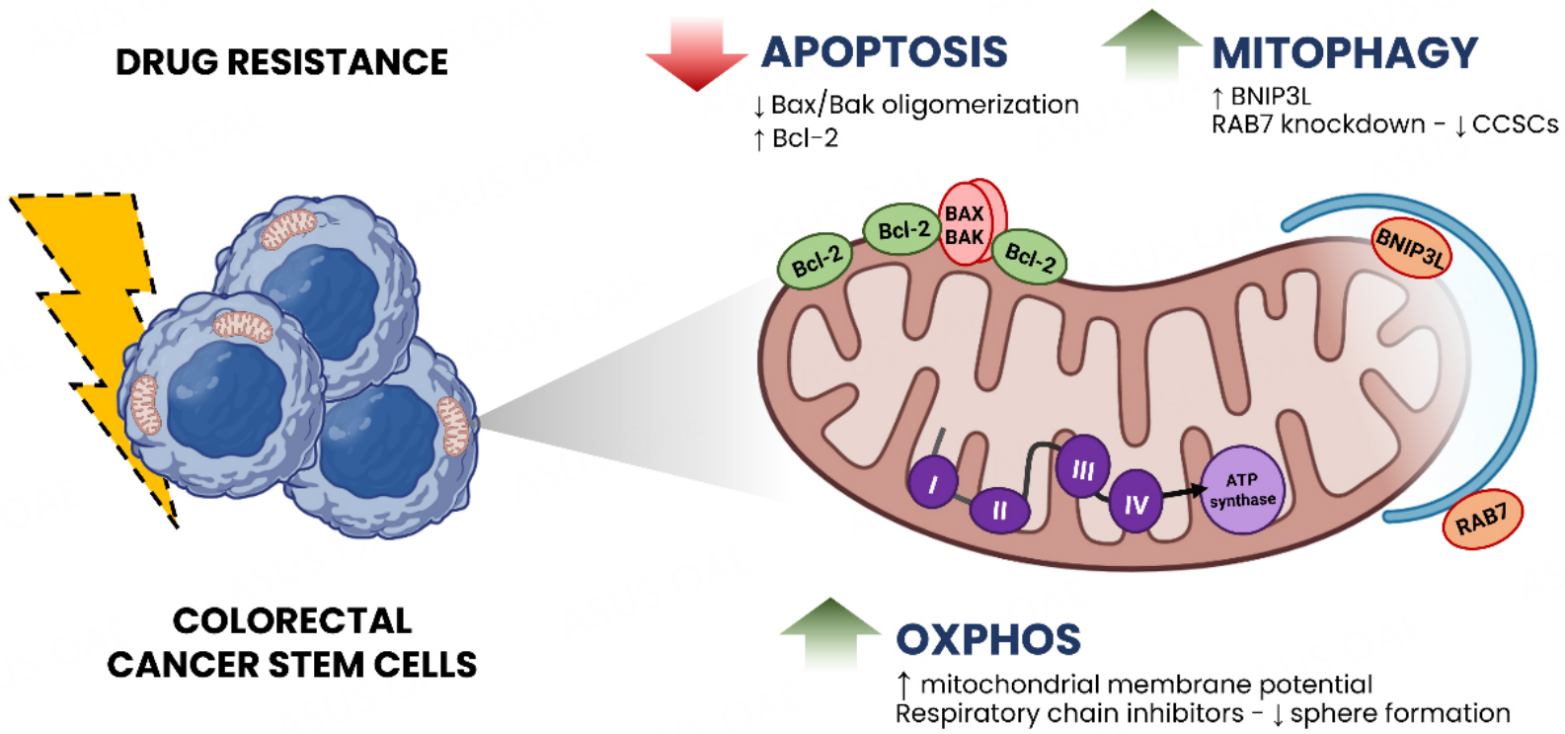
Mitochondria involvement in colorectal cancer stem cells and drug resistance. (1) Reduced apoptosis due to lower Bax/Bak oligomerization and higher Bcl-2 expression. (2) Enhanced mitophagy with the participation of RAB7 and higher BNIP3L expression. (3) Addiction to OXPHOS with high mitochondrial membrane potential. The vectors used in the figure are from BioRender©. BNIP3L: BCL2 Interacting Protein 3 Like; CCSCs: colorectal cancer stem cells; OXPHOS: oxidative phosphorylation; RAB7: Ras-related protein 7.

In CRC, there is an intimate relationship between CSCs and mitochondria in drug resistance scope [Figure 1]. Regarding the apoptosis mitochondrial pathway, recent data ensure that anti-apoptotic protein overexpression is vital for CCSCs chemoresistance. Purushothaman *et al.*^[75]^ showed that untreated CD133^+^ CCSCs (HCT116) expressed lower Bax/Bak oligomerization and higher Bcl-2 in comparison to Ruthenium(II) complex 1-treated group. In agreement with these findings, Colak *et al.*^[76]^ observed that CCSCs have decreased mitochondrial priming, accumulating anti-apoptotic proteins and inducing chemoresistance instead of differentiated CRC cells. In contrast, CCSCs incubated with WEHI-539, a specific inhibitor of Bcl-xl, were more sensitive to oxaliplatin, indicating that the mitochondrial apoptosis pathway is a great potential target for drug-resistant CCSCs. Ramesh *et al.*^[77]^ demonstrated that treatment with a BCL-XL inhibitor (A-1155463) increased the percentage of cells with active caspase-3 in Wnt-high CCSCs, indicating cell death, while BCL-2 (ABT-199) and MCL-1 (AZD5991) inhibitors did not promote this effect. These data suggest that CCSCs may depend only on BCL-XL and not on BCL-2 and MCL-1, indicating the therapeutic potential of inhibiting BCL-XL activity.

### Mitophagy rates

Mitophagy has also been assessed in CCSCs as it is another important mechanism for therapy resistance in cancer. Takeda *et al.*^[78]^ demonstrated that mitophagy was enhanced among CCSCs compared to non-CCSCs, and the knockdown of RAS-Associated proteins (RAB5 and RAB7) diminished the CD44v9^+^/CD133^+^ CCSCs population. Yan *et al.*^[79]^ showed that CD44^+^/CD133^+^ CCSCs (HCT8 cell lineage) were more resistant to doxorubicin treatment and expressed higher levels of BNIP3L, a mitophagy-related protein concerning parental cells. Instead, when CCSCs were treated with siRNA for BNIP3L, they became more sensitive to doxorubicin, suggesting that mitophagy is a major strategy of CCSCs to escape from death.

### Oxidative phosphorylation addiction

Regarding metabolism, Denise *et al.*^[80]^ showed that 5-FU resistant colorectal HT29 cancer cells are addicted to OXPHOS while expressing more CD133 and forming more spheres, a 3D cell culture, *in vitro*. The coadministration of 5-FU and respiratory chain complex inhibitors led to a shrinkage of spheres. Corroborating to these findings, Song *et al.*^[81]^ demonstrated that CD133^+^/CD44^+^/Lgr5^+^ CCSCs were highly dependent on OXPHOS and presented high mitochondrial membrane potential. Besides, it was observed that the incubation with 5-FU did not alter ATP levels of CD133^+^ CCSCs compared to non-CCSCs. Combinate treatment of 5-FU and antimycin A, an inhibitor of complex III from the respiratory chain, promoted cell death along with high expression of cleaved caspase 3. Thus, the addiction to OXPHOS appears to support CCSCs phenotype.

## CONCLUSION

Colorectal cancer remains a disease of great concern as therapy resistance remains a remarkable challenge. In this scenario, cancer stem cells are well-known contributors, and their mitochondria exhibited a valuable role in providing it. A major mechanism for chemotherapeutic avoidance in CRC is an enhancement in anti-apoptotic protein expression among CSCs, a higher mitophagy rate and a reliance on OXPHOS.

In this concern, studies involving approaches such as patient-derived xenografts and organoids should be conducted to expand the understanding of the correlation of CCSCs and their mitochondria, regarding energetic metabolism, apoptosis pathway, especially mitophagy, providing a better picture of the heterogeneity intrinsic to CRC and its chemoresistance. The literature also lacks studies about mitochondria morphology in CCSCs in drug resistance context, which is a potential topic for new remarks. Moreover, new chemotherapy strategies targeting mitochondria must be studied and developed aiming at the elimination of CCSCs and their great contribution to CRC chemotherapy resistance.

## DECLARATIONS

### Copyright

© The Author(s) 2023.
